# Evidence for Directed Evolution of Larger Size Motif in *Arabidopsis thaliana* Genome

**DOI:** 10.1100/2012/983528

**Published:** 2012-05-03

**Authors:** Rajesh Mehrotra, Amit Yadav, Purva Bhalothia, Ratna Karan, Sandhya Mehrotra

**Affiliations:** ^1^Department of Biological Sciences, Birla Institute of Technology and Science, Pilani, Rajasthan 333031, India; ^2^Department of Plant Environmentand Soil Sciences, Louisiana State University, Baton Rouge, LA 70894, USA

## Abstract

Transcription control of gene expression depends on a variety of interactions mediated by the core promoter region, sequence specific DNA-binding proteins, and their cognate promoter elements. The prominent group of *cis* acting elements in plants contains an ACGT core. The *cis* element with this core has been shown to be involved in abscisic acid, salicylic acid, and light response. In this study, genome-wide comparison of the frequency of occurrence of two ACGT elements without any spacers as well as those separated by spacers of different length was carried out. In the first step, the frequency of occurrence of the *cis* element sequences across the whole genome was determined by using BLAST tool. In another approach the spacer sequence was randomized before making the query. As expected, the sequence ACGTACGT had maximum occurrence in *Arabidopsis thaliana* genome. As we increased the spacer length, one nucleotide at a time, the probability of its occurrence in genome decreased. This trend continued until an unexpectedly sharp rise in frequency of (ACGT)N25(ACGT). The observation of higher probability of bigger size motif suggests its directed evolution in *Arabidopsis thaliana* genome.

## 1. Introduction

Gene expression in eukaryotic organisms has been a topic of great interest. Careful regulation and recruitment of transcription factors (TFs) to *cis* regulatory elements in promoter regions lead to generation of specificity and diversity [1] in genetic regulation. Promoters are arrays of *cis* regulatory elements present upstream of a gene arranged with other specific *cis *elements. At present 469 *cis *elements have been reported in the plant *cis* regulatory element (PLACE) database. The prominent group of *cis* acting elements in plants contains an ACGT core. Several *cis* elements with this core have been shown to be responding to abscisic acid [2–4], salicylic acid [5], and light signals [6]. It has been reported by Foster et al. [7] that bZIP class of transcription factors binds to this core motif. In an elegant study Krawczyk et al. [8] showed deletion of two base pairs between activator sequence-1 (as1) palindromes does not affect binding of activator sequence binding factor (ASF-1) and TGA factors (which binds to TGACG sequence), whereas insertion decreases factor binding *in vitro*. In their study the distance between palindromic centers was 12 base pairs. Mehrotra et al. [9, 10] have shown that this motif functions even when they are placed out of the native context. R. Mehrotra and S. Mehrotra [11] have shown that promoter activation by ACGT in response to salicylic and abscisic acids is differentially regulated by the spacing between these motifs. It contributes synergistically to gene expression by stabilising the transcription complex formed on minimal promoter [10]. The present study is an extension of aforementioned work. In this study, genome-wide comparison of the frequency of occurrence of two ACGT elements without any spacers and also separated by spacers of different lengths was done. Based on the data obtained we report that there is a directed evolution of bigger size of motif in the *Arabidopsis thaliana* genome.

## 2. Materials and Methods

The objective was to find out the frequency of the recurring sequences and then use these recurring sequences with a random minimal promoter to predict transcription factors likely to interact with them.

The genomic sequence database of *Arabidopsis thaliana* at http://www.arabidopsis.org/ (The *Arabidopsis *Information Resource, TAIR) was analyzed using software BLASTn (available at NCBI website). All sequences were run in BLASTn against whole *Arabidopsis thaliana* genome to find their frequency of occurrence. Accession numbers of *Arabidopsis thaliana* chromosomes are as follows: chromosome 1: NC_003070.9, chromosome 2: NC_003071.7, chromosome 3: NC_003074.8, chromosome 4: NC_003075.7, and chromosome 5: NC_003076.8.

Randomization of the sequence was carried out using SHUFFLE program [12]. Different sequences obtained are listed in Table 1. In the next step we found the transcription factors binding to these *cis* elements separated by different length of nucleotides. A 139 bp long minimal promoter *Pmec* [13] was used in this study. The minimal promoter sequence as shown below was suffixed to the sequences shown in Table 1;

TCACTATATATAGGAAGTTCATTTCATTTGGAATGGACACGTGTTGTCATTTCTCAACAATTACCAACAACAACAAACAACAAACAACATTATACAATTACTATTTACAATTACATCTAGATAAACAATGGCTTCCTCC.

These extended sequences were used in JASPAR core database [14] to scan for transcription factors and then these TFs were crosschecked with results obtained from CONSITE [15].

## 3. Results and Discussion

### 3.1. Promoters with Greater Length between ACGT Motifs Are More Frequent

It has been reported that ACGT *cis* elements function even when they are placed out of native sequence context [9, 10]. When the distance of separation between two ACGT elements are 5 base pairs, and 10 base pairs, they are induced in response to salicylic acid (SA) and abscisic acid (ABA), respectively. Interestingly, SA mimics biotic stress response and ABA mimics abiotic stress response in plants and thus is of great interest to plant biologists. Paixão and Azevedo [16] showed that multiplicity of *cis* element evolved through transitional forms showing redundant* cis* regulation. In this study, when the frequency of occurrence of two ACGT elements without any spacers and also separated by the spacer of different lengths was observed, we found that the total frequency of occurrence of two ACGT element in tandem is 1885 (Table 1), while the e value was same for all alignments obtained on a particular chromosome. When two ACGT elements were separated by spacer of 5, 10, and 25 nucleotides their frequency of occurrence was 72, 39, and 62, respectively. An unexpectedly high frequency of occurrence was observed when two ACGT elements were separated by 25 base pairs. According to the rule of probability the frequency of two ACGT elements separated by 25 base pairs should be less than when they are separated by 10 base pairs or lesser. Hobo et al. [17] have earlier reported that in ABA responsive promoters the distance between ACGT elements is 30 base pairs. To address this discrepancy in the data obtained, we randomized the spacer sequence keeping the ACGT motif unchanged. The logic of this randomization was to identify how important is the distance between the binding sites for transcription factors. After randomization of the spacer there was a drop in the frequency of occurrence to 23, 14, and 21 from 72, 39, and 62 for (ACGT)_N5_(ACGT), (ACGT)_N10_(ACGT), and (ACGT)_N25_(ACGT), respectively. This means that along with the distance between binding motifs there has been a positive selection for the sequence of the spacer in transcriptional regulation. In the next step we completely randomized the sequence and we observed that there is a drop in frequency of occurrence of two ACGT elements when separated by 10 and 25 base pairs while there was an unexpected increase in the frequency when ACGT elements were separated by five base pairs. This happened because randomization generated a motif that has been positively selected in evolution.

### 3.2. A and G Are the Preferred Bases

We increased the spacer length one residue at a time and looked for the frequency of each resultant sequence in the database. As shown in Table 2, there has been preference for A and G in the spacer region between two ACGT sequences.

### 3.3. Increasing Spacing between Motifs Increases Transcription Factor Binding Sites

Potential transcription factor binding sites for all experimental sequences when predicted using JASPAR CORE software and subsequently crosschecked with CONSITE revealed the minimal promoter sequence to be possessing 35 potential TF binding sites (Table 3, MPS). Interestingly the sequence ACGT as such has no site for binding of transcription factors but when minimal promoter is suffixed to it, an extra site for squamosa is generated and the total transcription factor binding site increases from 35 to 36 in minimal promoter alone (Table 3, (ACGT)(MPS)). When two ACGT elements in tandem are placed over minimal promoter sequence no extra site for binding of transcription factor is generated (Table 3, (ACGT)_2_(MPS)). However, when ACGT elements are separated by five base pairs (Table 3, (ACGT)_N5_(ACGT)(MPS)), four additional transcriptional binding sites are generated while ATHB-5 binding site which existed in the earlier cases is lost. The new sites generated are for transcription factors bzip9-10, EmBP-1, myb.Ph3, and TGA1a. Placement of two ACGT elements separated by 10 base pairs, however, resulted in loss of one myb.Ph3 site and the total transcriptional binding site decreased to 38 (Table 3, (ACGT)_N10_(ACGT)(MPS)). In case when ACGT elements are separated by 25 base pairs followed by minimal promoter an additional site for ARR10 and dof3 was generated (Table 3, (ACGT)_N25_(ACGT)(MPS)).

Based on the data obtained in this study, we report here that there has been directed evolution of bigger size of the motif in the *Arabidopsis thaliana* genome.

## 4. Conclusions

The central question in promoter evolution is to know how does *cis* regulatory element multiplicity evolved. The promoter regions of many genes contains multiple binding sites for the same transcription factor. Multiplicity may have evolved through transitional forms showing redundant *cis* regulation. In this paper, we focused on multiplicity of ACGT *cis *element and the distances between them which occurs in natural promoters. We found that ACGT element separated by 25 base pairs is more frequent than those by 10 base pairs which is against the law of probability. It signifies that under some evolutionary forces this interval was favoured since this distance may cause changes in the level of gene expression or in its robustness against variation in transcription factor concentration. Selection for different levels of expression of certain genes in certain environment could, over time, generates a positive association between *cis *element multiplicity and expression level.

## Figures and Tables

**Table 1:** Frequency of occurrence of the various promoter sequences in which spacer sequence length between two ACGT palindromes is gradually increased from 5 to 25 nucleotides.

	Cis element	Chromosome 1	Chromosome 2	Chromosome 3	Chromosome 4	Chromosome 5	Total
(ACGT)_ 2_	ACGTACGT	469	312	367	327	410	1885
(ACGT)_ 8_	ACGTACGTACGTACGTACGTACGTACGTACGT	70	31	12	28	59	200
(ACGT)_N5_(ACGT)	ACGTGGCTAACGT	16	11	13	13	19	72
(ACGT)_N10_(ACGT)	ACGTGGCTATGGCGACGT	8	5	10	4	12	39
(ACGT)_N25_(ACGT)	ACGTGGCTATGGCGGAGCAAGATTCACTCACGT	15	12	13	9	13	62
(ACGT)_RN5_(ACGT)	ACGT–GCTAG–ACGT	7	5	5	2	4	23
(ACGT)_RN10_(ACGT)	ACGT–TGGGGCCGAT–ACGT	2	2	4	3	3	14
(ACGT)_RN25_(ACGT)	ACGTAGACACGTTGGGGGAACTTACTGCCACGT	3	1	7	5	5	21
(ACGT)_RN25_(ACGT)	ACGT-ATATGAGATCGGCGCTTCACGGAGC-ACGT	4	14	6	4	4	32
(ACGT)_N5_(ACGT) randomized	GGAATCCTTGGCA	41	24	30	19	23	137
(ACGT)_N10_(ACGT) randomized	GCGGGCTATCGGTAGCAT	2	5	2	0	1	10
(ACGT)_N25_(ACGT) randomized	TAAGGCTTAGCCACGCTTAGGGTGTGAGCACAC	6	6	3	0	3	18
(TGCA)_N25_(TGCA)	TGCAGGCTATGGCGGAGCAAGATTCACTCTGCA	13	12	9	12	9	55

N5, N10, N25 denote sequence length between two ACGT palindromes. RN5, RN10, RN25—signify only spacer sequence being randomized. (ACGT)_ N__(ACGT) randomized—signify complete sequence being randomized.

**Table 2:** Frequency of occurrence of nitrogenous bases when spacer sequence length between two ACGT palindromes is gradually increased from 5 to 25 nucleotides.

		A	C	G	T	Seq. used	Gap	Count
(ACGT)_N5_(ACGT)	ACGTGGCT_ACGT	72	42	33	34	72	5	690
(ACGT)_N6_(ACGT)	ACGTGGCTA_ACGT	98	65	45	44	44	6	611
(ACGT)_N7_(ACGT)	ACGTGGCTAT_ACGT	92	91	77	80	77	7	824
(ACGT)_N8_(ACGT)	ACGTGGCTATG_ACGT	97	30	64	55	64	8	852
(ACGT)_N9_(ACGT)	ACGTGGCTATGG_ACGT	39	32	22	32	32	9	602
(ACGT)_N10_ (ACGT)	ACGTGGCTATGGC_ACGT	34	36	39	66	39	10	600
(ACGT)_N11_(ACGT)	ACGTGGCTATGGCG_ACGT	36	23	38	29	38	11	681
(ACGT)_N12_(ACGT)	ACGTGGCTATGGCGG_ACGT	56	54	65	45	56	12	638
(ACGT)_N13_(ACGT)	ACGTGGCTATGGCGGA_ACGT	78	50	77	59	77	13	652
(ACGT)_N14_(ACGT)	ACGTGGCTATGGCGGAG_ACGT	86	53	96	52	53	14	841
(ACGT)_N15_(ACGT)	ACGTGGCTATGGCGGAGC_ACGT	56	67	44	66	56	15	709
(ACGT)_N16_(ACGT)	ACGTGGCTATGGCGGAGCA_ACGT	60	34	52	34	60	16	843
(ACGT)_N17_(ACGT)	ACGTGGCTATGGCGGAGCAA_ACGT	39	41	42	39	42	17	830
(ACGT)_N18_(ACGT)	ACGTGGCTATGGCGGAGCAAG_ACGT	49	47	58	48	49	18	719
(ACGT)_N19_(ACGT)	ACGTGGCTATGGCGGAGCAAGA_ACGT	50	38	49	44	44	19	695
(ACGT)_N20_(ACGT)	ACGTGGCTATGGCGGAGCAAGAT_ACGT	34	30	44	37	37	20	821
(ACGT)_N21_(ACGT)	ACGTGGCTATGGCGGAGCAAGATT_ACGT	36	40	42	43	40	21	717
(ACGT)_N22_(ACGT)	ACGTGGCTATGGCGGAGCAAGATTC_ACGT	53	42	42	46	53	22	726
(ACGT)_N23_(ACGT)	ACGTGGCTATGGCGGAGCAAGATTCA_ACGT	91	55	60	61	55	23	771
(ACGT)_N24_(ACGT)	ACGTGGCTATGGCGGAGCAAGATTCAC_ACGT	77	64	57	53	53	24	1171
(ACGT)_N25_(ACGT)	ACGTGGCTATGGCGGAGCAAGATTCACT_ACGT	76	62	58	69	62	25	708

**Table 3:** Alterations in transcription factor binding sites when spacer sequence length between two ACGT palindromes is gradually increased from 5 to 25 nucleotides.

	Minimal promoter sequence (MPS)	(ACGT)	(ACGT)(MPS)	(ACGT)_2_(MPS)	(ACGT)_N5_(ACGT)(MPS)	(ACGT)_N10_(ACGT)(MPS)	(ACGT)_N25_(ACGT)(MPS)
Model name	Frequency						

ARR10	0	0	0	0	0	0	1
AGL3	2	0	2	2	2	2	2
ATHB-5	1	0	1	2	1	1	1
bZIP910	0	0	0	0	1	1	1
Dof3	1	0	1	1	1	1	2
EmBP-1	2	0	2	1	2	2	2
Gamyb	5	0	5	5	5	5	5
HAT5	2	0	2	2	2	2	2
HMG-1	6	0	6	6	6	6	6
HMG-I/Y	6	0	6	6	6	6	6
id1	5	0	5	5	5	5	5
myb.Ph3	1	0	1	1	2	1	1
PEND	1	0	1	1	1	1	1
squamosa	2	0	3	3	3	3	3
TGA1A	1	0	1	1	2	2	2
	**35**	**0**	**36**	**36**	**39**	**38**	**40**
